# Optimization of air-blast drying process for manufacturing *Saccharomyces cerevisiae* and non-*Saccharomyces* yeast as industrial wine starters

**DOI:** 10.1186/s13568-016-0278-9

**Published:** 2016-11-08

**Authors:** Sae-Byuk Lee, Won-Seok Choi, Hyun-Jung Jo, Soo-Hwan Yeo, Heui-Dong Park

**Affiliations:** 1 School of Food Science and Biotechnology, Kyungpook National University, 80 Daehakro, Daegu, 41566 South Korea; 2 Department of Fermentation Biotechnology, Kyungpook National University, 80 Daehakro, Daegu, 41566 South Korea; 3 Fermented Food Science Division, Depertment of Agro-food Resources, NIAS, RDA, 166, Nongsaengmyeong-ro, Iseo-myeon, Wanju Gun, Jeollabuk-do, 55365 South Korea

**Keywords:** Wine yeast, Air-blast drying, Survival rate, Excipient, Lactomil

## Abstract

Wine yeast (*Saccharomyces cerevisiae* D8) and non-*Saccharomyces* wine yeasts (*Hanseniaspora uvarum* S6 and *Issatchenkia orientalis* KMBL5774) were studied using air-blast drying instead of the conventional drying methods (such as freeze and spray drying). Skim milk—a widely used protective agent—was used and in all strains, the highest viabilities following air-blast drying were obtained using 10% skim milk. Four excipients (wheat flour, nuruk, artichoke powder, and lactomil) were evaluated as protective agents for yeast strains during air-blast drying. Our results showed that 7 g lactomil was the best excipient in terms of drying time, powder form, and the survival rate of the yeast in the final product. Finally, 7 types of sugars were investigated to improve the survival rate of air-blast dried yeast cells: 10% trehalose, 10% sucrose, and 10% glucose had the highest survival rate of 97.54, 92.59, and 79.49% for *S. cerevisiae* D8, *H. uvarum* S6, and *I. orientalis* KMBL5774, respectively. After 3 months of storage, *S. cerevisiae* D8 and *H. uvarum* S6 demonstrated good survival rates (making them suitable for use as starters), whereas the survival rate of *I. orientalis* KMBL5774 decreased considerably compared to the other strains. Air-blast dried *S. cerevisiae* D8 and *H. uvarum* S6 showed metabolic activities similar to those of non-dried yeast cells, regardless of the storage period. Air-blast dried *I. orientalis* KMBL5774 showed a noticeable decrease in its ability to decompose malic acid after 3 months of storage at 4 °C.

## Introduction

Wine is one of the oldest fermented foods in history and is produced as a result of complicated interplay between the metabolic reactions of various microorganisms such as yeast and lactic acid bacteria (Zagorc et al. 2001). Wine yeast, *Saccharomyces cerevisiae*, has been used to make wine with high stability because of its high ethanol tolerance and ability to inhibit bacteria and other undesirable microorganisms during the fermentation process (Casey and Ingledew 1986; Philliskirk and Young 1975). On the other hand, non-*Saccharomyces* yeasts, which grow during the initial stages of fermentation, affect the taste and aroma of wine, suggesting that suitable co-fermentation using *Saccharomyces* yeasts mixed with non-*Saccharomyces* yeast is an important factor in making wine of high quality (Ciani and Maccarelli 1998; Rojas et al. 2001; Jolly et al. 2006; Esteve-Zarzoso et al. 1998).

Several Korean wine-makers have widely utilized *S. cerevisiae* Fermivin from Netherlands, *S. cerevisiae* W-3 from Japan, and *S. cerevisiae* EC1118 from Canada because these strains can be handled conveniently and offer reliable starter quality. Although most Korean wine has been made using these imported yeast starters, several studies have reported that indigenous yeasts can also contribute to making distinctive wines based on the grape cultivar and the geographical region (Heard and Fleet 1985; Mercado et al. 2007; Querol et al. 1992; Schütz and Gafner 1993; Hong and Park 2013).

The Campbell Early grape, which is the most dominant cultivar in Korea, has a high malic acid content due to early harvesting for enhancing grape color. High malic acid content lowers the quality of Korean wine due to high acidity, which has resulted in the poor competitive value of indigenously manufactured wine against imported wine (Kim et al. 1999; Lee et al. 2016). For this reason, isolating and utilizing indigenous yeasts instead of imported yeast starters are necessary to make Korean wine competitive. Developing optimal industrial starter cultures for winemaking is essential for increasing the prevalence of indigenous Korean yeast starter products. Previously, *S. cerevisiae* D8, *Hanseniaspora uvarum* S6, and *Issatchenkia orientalis* KMBL5774 were isolated from Korean Campbell Early grape cultivar and their biological and physiological characteristics were studied. Kim et al. (2013b) reported that wine fermented by *S. cerevisiae* D8 had higher color and taste scores compared to the wine fermented by *S. cerevisiae* W-3. Hong and Park (2013) described that wine fermented by *H. uvarum* S6 (previously SS6) showed slower fermentation rate but had higher organic acid content and sensory evaluation scores compared to wine fermented by *S. cerevisiae* W-3. Seo et al. (2007) and Kim et al. (2008) reported that *I. orientalis* KMBL5774 could degrade malic acid during alcohol fermentation, and co-fermentation with *I. orientalis* KMBL5774 and *S. cerevisiae* W-3 resulted in better color, flavor, and taste compared to the fermentation using only *S. cerevisiae* W-3.

The most important factors for developing microbial starters include maintenance of cell viability, capacity for long-term storage and the drying method used. Several studies have utilized freeze-drying (Lodato et al. 1999; Ale et al. 2015; Abadias et al. 2001a), fluidized bed drying (Bayrock and Ingledew 1997), and spray drying (Luna-Solano et al. 2005; Isono et al. 1995) to make starter products. Freeze-drying is disadvantageous owing to the high expenses incurred, and fluidized bed drying and spray drying are not suitable due to low viability in the starter cultures induced by the high temperature during drying. In contrast, air-blast drying can lower the cost fivefold, result in comparatively less cell damage, as well as provide easier control of moisture in the starter compared with other drying methods (Santivarangkna et al. 2007). Even though air-blast drying has many advantages for making yeast starters, only a few studies related to the air-blast dried yeast have been attempted. Similar to freeze-drying, the selection of protective agents is very important in air-blast drying because intracellular accumulation of the appropriate solutes is related to strain survival following air-blast drying (Kets et al. 1996; Champagne et al. 2012). Suitable agents can protect the proteins and membranes of the microorganisms (Leslie et al. 1995; Champagne and Gardner 2001).

In this study, we aimed to optimize the development of *Saccharomyces* and non-*Saccharomyces* yeast starters at the industrial level using air-blast drying, instead of the conventionally used freeze-drying method, as well as using various types of excipients and sugars at different concentrations to enhance the survival rate of air dried-yeast cells. Furthermore, the long-term storage properties of each dried-yeast strain and the metabolic activity of air-blast dried yeast cells during storage at 4 °C were also investigated.

## Materials and methods

### Strains, media, and culture conditions

*Saccharomyces cerevisiae* D8 (KACC 93245P), *H. uvarum* S6 (KACC 93248P) and *I. orientalis* KMBL5774 (KACC 93124P) isolated from the Korean grape cultivar were used in this study (Hong and Park 2013; Kim et al. 2013a; Seo et al. 2007). Each strain was cultured at 30 °C with shaking (150 rpm) in sterilized YPD media composed of 1% yeast extract, 2% bacto-peptone, and 2% glucose and the cells were harvested for making the starters. All strains were stored at −70 °C in 20% glycerol until they were used for the experiments.

### Protective agent conditions

Skim milk (5 and 10%) and 7 sugars (5 and 10% of glucose, fructose, lactose, maltose, raffinose, sucrose, and trehalose) were used to evaluate the survival rate of air-blast dried yeast cells. All protective solutions, including skim milk and sugars were sterilized at 121 °C for 15 min before experiments. Four kinds of excipients—wheat flour (CJ Cheiljedang Corp., Seoul, Korea), nuruk (Songhak Agri. Corp., Gwangju, Korea), artichoke powder and lactomil (composed of lactose 89% and maltodextrin 11%; Seo Kang Dairy & Food Co., LTD, Sacheon, Korea)—were used to process the yeast starters into an appropriate powdered form. Artichoke was obtained from Gimcheon, Korea, and it was processed by lyophilization and grinding to be converted into powdered form. All excipients were added at quantities of 2 g (lactomil amounts ranged from 2 to 8 g) to compare their protective ability and availability as a starter product in powdered form for each strain of the dried yeast. All excipients were used with the yeast pellet directly.

### Air-blast drying process

Each yeast strain was cultured in 100 mL YPD broth and incubated at 30 °C for 16 h. After culturing, yeast cells were harvested by centrifugation (3578×*g* for 10 min) and rinsed twice in a 0.85% NaCl solution. The pellet was mixed with 2 g of various excipients such as wheat flour, nuruk, and artichoke powder, and lactomil (2–8 g) as well as 1 mL protective agent solutions consisting of the skim milk and sugars. The mixed yeast cell pellets were dried using Clear Air Oven (HB-509C, HanBaek, Bucheon, Korea) at 37 °C until the moisture content of dried yeast starter was <10%. After air-blast drying, the samples were immediately analyzed to determine their moisture content and survival rate, then stored at 4 °C for 3 months, after which their survival rate was determined.

### Measurement of cell viability and moisture content

After air-blast drying, each sample was reconstituted to its original volume with distilled water for rehydration. Then, the serially diluted samples were spread on YPD agar plates and incubated at 30 °C for 24 h. The white colonies that formed on YPD agar were counted. The survival rate of each sample was calculated as (%) survival = (N/N_0_) × 100, where N represents the number of viable cell count after air-blast drying (cfu mL^−1^) and N_0_ represents the number of viable cell count before air-blast drying (cfu mL^−1^). Moisture content of dried yeast starters was measured by determining the weight loss after 10 h at 105 °C (AOAC 1990).

### Morphology of air-blast dried yeast cells

The morphologies of air-blast dried *Saccharomyces* and non-*Saccharomyces* yeast cells were observed by scanning electron microscopy (SEM), as described by Hongpattarakere et al. (2013). The air-blast dried sample was affixed to “stubs” using double-sided metallic adhesive tape and then coated with gold using sputter coater (WI-RES-Coater-001). The morphology of the sample was observed under a SU8220 scanning electron microscope (Hitachi, Tokyo, Japan) that was operated at an accelerating voltage of 10 kV. Images were obtained under 2000× magnification.

### Metabolic activities of air-blast dried yeast cells

Metabolic activities of yeast cells stored for 0–3 months after air-blast drying were analyzed and non-dried yeast cells were used as the control. Air-blast dried *S. cerevisiae* D8 and *H. uvarum* S6 were incubated in 100 mL YPD broth containing 20% glucose (yeast extract 10 g L^−1^, peptone 20 g L^−1^, and glucose 200 g L^−1^) at 30 °C with shaking (150 rpm) to measure the glucose fermentation rate. A water trap apparatus containing conc. H_2_SO_4_ was attached to the top of each flask to trap water evaporated from the flask during the fermentation. The amount of CO_2_ produced was directly measured as the decrease in the weight of the whole flask. The fermentation ratio was expressed as the percentage of the amount of CO_2_ produced per the theoretical CO_2_ production from the glucose due to the ethanol fermentation (Jung and Park 2005). Air-blast dried *I. orientalis* KMBL5774 was incubated in 10 mL YPD broth containing 2% malic acid at 30 °C with shaking (150 rpm) to measure the malic acid decomposition rate. Malic acid content was determined using the  l-Malic Acid Assay Kit (K-LMALR; Megazyme, Wicklow, Ireland) (Lee et al. 2016).

### Statistical analysis

All experiments were carried out in at least triplicates and the results were analyzed using the Statistical Package for the Social Sciences (SPSS, version 12.0 for Windows, Chicago, IL, USA) in order to obtain average and standard deviations. Significance was determined to be *p* < 0.05 using one-way analysis of variance (ANOVA), followed by Duncan’s multiple range test.

## Results

### Effect of skim milk on the survival rate of air-blast dried yeast cells

**Fig. 1:**
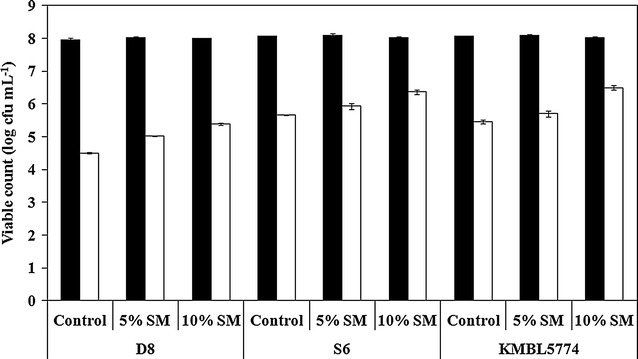
Viable yeast cell counts with 5 and 10% skim milk before (*filled squares*) and after (*empty squares*) air-blast drying for 2 h. All data are expressed as the mean ± SD (n = 3)

Skim milk is typically used as a protective agent to protect cell membrane while drying microbial strains. It has been suggested that milk proteins may cover the cells to prevent damage (Abadias et al. 2001b). In this study, the protective effect of skim milk on air-blast dried cells was investigated. For this, 5–10% skim milk solutions were mixed with centrifuged yeast cells and the mixed cells were air-blast dried at 37 °C for 2 h until dried cells were obtained in the appropriate powdered form. The survival rates of all air-blast dried yeast cell strains mixed with skim milk increased in a dose-dependent manner compared to that of the control (Fig. 1). When 10% skim milk was mixed with the dried yeast cells, the viable count of *S. cerevisiae* D8, *H. uvarum* S6, and *I. orientalis* KMBL5774 increased to approximately 0.89, 0.71, and 1.03 log cfu mL^−1^, respectively. Thus, 10% skim milk was utilized in subsequent experiments.

### Effect of various excipients on the survival rate and moisture content of air-blast dried yeast cells

**Fig. 2:**
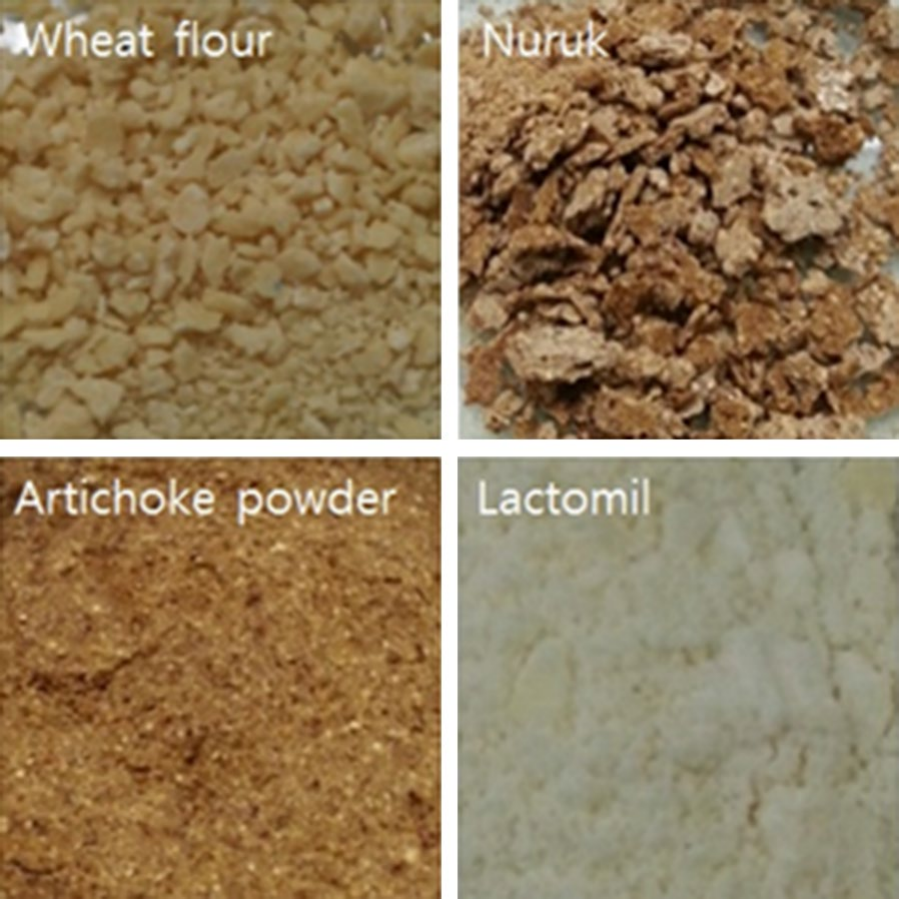
Images of air-blast dried yeast cells mixed with wheat flour, nuruk, artichoke power, and lactomil. All excipients were added at a concentration of 2 g and air-blast dried until moisture content of samples was <10%

The selection of the excipient is very important in generating a stable powdered form and shape for improving the stability and quality of the final product (Georgetti et al. 2008). In this study, four excipients (wheat flour, nuruk, artichoke powder, and lactomil) were utilized to prevent cell membrane damage caused by the drying environment and the survival rates and moisture contents were determined. All samples were air-blast dried until the moisture content reached <10%. The air-blast dried yeast samples mixed with wheat flour showed the longest drying time of 7 h and the lowest survival rate of 0.27–1.39%, whereas the samples mixed with lactomil showed the shortest drying time of 2.5 h and the highest survival rate of 1.01–3.40% (Table 1). On analysis of the shape of the powdered form obtained by adding various excipients, we found that the samples mixed with wheat flour and nuruk showed a lump form after air-blast drying, and the samples mixed with artichoke powder and lactomil could be easily collected due to their impalpable powdered form (Fig. 2). Based on the survival rate, drying time, and the properties of the powdered form of air-blast dried yeast products, 2 g lactomil was considered as the most suitable excipient for making yeast starters by air-blast drying. Based on the results of the yeast samples added to various excipients, the survival rates of air-blast dried yeast cells in relation to the amount of lactomil added were investigated (Table 2). As the amount of lactomil added was increased, drying time for each sample with <10% moisture content was reduced and the survival rate of air-blast dried yeast cells increased in a dose-dependent manner until the addition of 7 g lactomil. In case of *S. cerevisiae* D8, the survival rate of the sample added to 7 g lactomil was measured as 59.12%, which was higher than the survival rate of the sample added to 8 g lactomil. In case of *H. uvarum* S6 and *I. orientalis* KMBL5774, the survival rates measured for 7–8 g of lactomil were not significantly different. Although the drying time of the samples added to 8 g lactomil was 0.3 h shorter than that of the samples added to 7 g lactomil, after considering the excipient cost and the similar protective effect of 7–8 g of lactomil, 7 g was considered as the optimal amount of lactomil required for maintaining the viability of air-blast dried yeast cells. Therefore, subsequent experiments were carried out by adding 10% skim milk and 7 g lactomil to the yeast cells, followed by air-blast drying for 1.5 h.

**Table 1:** Effects of various excipients on the survival rate and moisture content of air-blast dried yeasts

Excipient	*S. cerevisiae* D8		*H. uvarum* S6		*I. orientalis* KMBL5774		Drying time (h)
	Survival rate (%)	Moisture content (%)	Survival rate (%)	Moisture content (%)	Survival rate (%)	Moisture content (%)	
Wheat flour	1.39 ± 0.04^d^	9.53 ± 0.30	0.27 ± 0.04^d^	9.21 ± 0.27	0.30 ± 0.02^c^	9.31 ± 0.20	7
Nuruk	2.23 ± 0.14^c^	9.86 ± 0.23	0.36 ± 0.03^c^	9.94 ± 0.19	0.33 ± 0.03^c^	9.90 ± 0.35	4
Artichoke powder	2.53 ± 0.08^b^	9.89 ± 0.38	0.61 ± 0.04^b^	9.50 ± 0.31	1.07 ± 0.08^b^	9.73 ± 0.23	6
Lactomil	3.40 ± 0.19^a^	9.42 ± 0.12	1.01 ± 0.06^a^	9.23 ± 0.16	1.65 ± 0.33^a^	8.96 ± 0.14	2.5

Different letters within the same column indicate significant difference (*p* < 0.05)

**Table 2:** Effects of the amount of lactomil added on the survival rate and moisture content of air blast dried yeasts

Lactomil (g)	*S. cerevisiae* D8		*H. uvarum* S6		*I. orientalis* KMBL5774		Drying time (h)
	Survival rate (%)	Moisture content (%)	Survival rate (%)	Moisture content (%)	Survival rate (%)	Moisture content (%)	
2	3.40 ± 0.19^e^	9.42 ± 0.12	1.01 ± 0.06^e^	9.23 ± 0.21	1.65 ± 0.33^c^	8.96 ± 0.15	2.5
3	5.35 ± 0.30^e^	8.37 ± 0.19	2.46 ± 0.59^e^	8.27 ± 0.13	3.11 ± 0.21^c^	7.85 ± 0.11	2.5
4	32.70 ± 2.37^d^	8.81 ± 0.24	6.33 ± 0.32^d^	9.70 ± 0.17	11.79 ± 0.84^b^	9.30 ± 0.21	2
5	39.31 ± 3.03^c^	9.52 ± 0.21	9.52 ± 0.42^c^	8.54 ± 0.15	13.66 ± 2.87^b^	8.81 ± 0.13	2
6	43.40 ± 3.27^c^	9.17 ± 0.18	17.18 ± 1.25^b^	9.68 ± 0.29	16.33 ± 2.86^b^	7.23 ± 0.09	2
7	59.12 ± 1.96^a^	8.88 ± 0.24	29.65 ± 1.77^a^	8.34 ± 0.14	23.71 ± 3.38^a^	8.48 ± 0.16	1.5
8	53.45 ± 4.75^b^	9.58 ± 0.31	29.60 ± 3.16^a^	9.84 ± 0.32	23.68 ± 3.42^a^	9.37 ± 0.22	1.2

Different letters within the same column indicate significant difference (*p* < 0.05)

### Effect of sugar additives as a protective agent on the survival rate of air-blast dried yeast cells

**Fig. 3:**
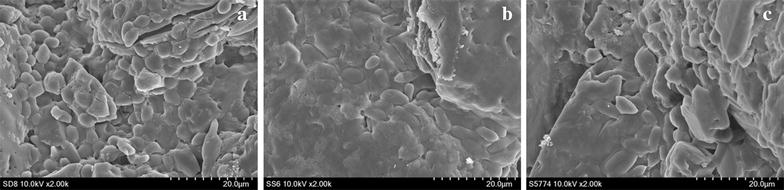
Images of air-blast dried yeast cells observed by a scanning electron microscope (SEM) at ×2000 magnification. 10% skim milk and 7 g lactomil were used in all samples. Ten percent trehalose, 10% sucrose, and 10% glucose were used as protective agents for *S. cerevisiae* D8 (**a**), *H. uvarum* S6 (**b**), and *I. orientalis* KMBL5774 (**c**), respectively

To determine the protective effect of sugar on air-blast dried yeast cells, the survival rate of air-blast dried yeast cells (depending on the type and concentration of sugars as protective agents with 10% skim milk) were investigated (Table 3). In case of *S. cerevisiae* D8, the addition of 10% sugars (except for fructose) resulted in a survival rate of >90%, and addition of 10% trehalose resulted in the highest survival rate of 97.54%. In case of *H. uvarum* S6, the high survival rates of all samples added to sugars resulted in considerably higher viability than that of samples with no sugar addition. All samples added to 10% sugars had higher survival rate than those added to 5% sugars; in particular, 10% sucrose resulted in the highest survival rate of 92.59%. In case of *I. orientalis* KMBL5774, most sugar additions did not show a significant increase of the survival Malic acid content was determined rate compared to no sugar addition, but addition of 10% glucose and 10% fructose noticeably increased its survival rate to 79.49 and 65.17%, respectively. The morphology of air-blast dried yeast cells was observed using SEM (Fig. 3). The SEM images showed that each yeast cell was coated with skim milk, sugar, and lactomil and the cells were densely accumulated, which suggest that protective agents and excipients protect yeast cells from the adverse drying environment.

**Table 3:** Effects of the type and concentration of various sugars on the survival rate of air-blast dried yeasts

Strains	Conc. (%)	Survival rate (%)						
		Glucose	Fructose	Lactose	Maltose	Raffinose	Sucrose	Trehalose
*S. cerevisiae* D8	5	95.07 ± 8.45^a^	64.31 ± 7.62^b^	84.00 ± 12.17^a^	88.80 ± 6.59^a^	93.39 ± 15.32^a^	94.38 ± 11.68^a^	96.13 ± 5.94^a^
	10	94.33 ± 6.66^a^	74.28 ± 7.07^ab^	91.20 ± 9.53^a^	94.03 ± 5.13^a^	94.52 ± 10.46^a^	93.73 ± 8.74^a^	97.54 ± 6.77^a^
*H. uvarum* S6	5	63.99 ± 5.81^b^	67.67 ± 1.06^b^	72.56 ± 8.75^ab^	67.20 ± 4.07^b^	68.54 ± 2.35^b^	69.66 ± 6.76^ab^	69.77 ± 1.55^ab^
	10	71.71 ± 4.78^ab^	75.59 ± 11.05^ab^	85.81 ± 4.98^ab^	78.76 ± 17.82^ab^	84.68 ± 9.30^ab^	92.59 ± 11.17^a^	73.38 ± 9.18^ab^
*I. orientalis* KMBL5774	5	41.89 ± 3.58 ^cd^	39.68 ± 5.99 ^cd^	43.04 ± 9.56 ^cd^	39.74 ± 4.62 ^cd^	41.03 ± 8.97 ^cd^	40.79 ± 2.63 ^cd^	44.30 ± 11.04 ^cd^
	10	79.49 ± 9.25^a^	65.17 ± 5.15^b^	36.71 ± 4.56^d^	54.17 ± 8.46^bc^	33.33 ± 4.62^d^	37.78 ± 3.39^d^	45.93 ± 6.06 ^cd^

Different letters within the same strains indicate significant difference (*p* < 0.05)

### Long-term storability of air-blast dried yeast starter products

**Fig. 4:**
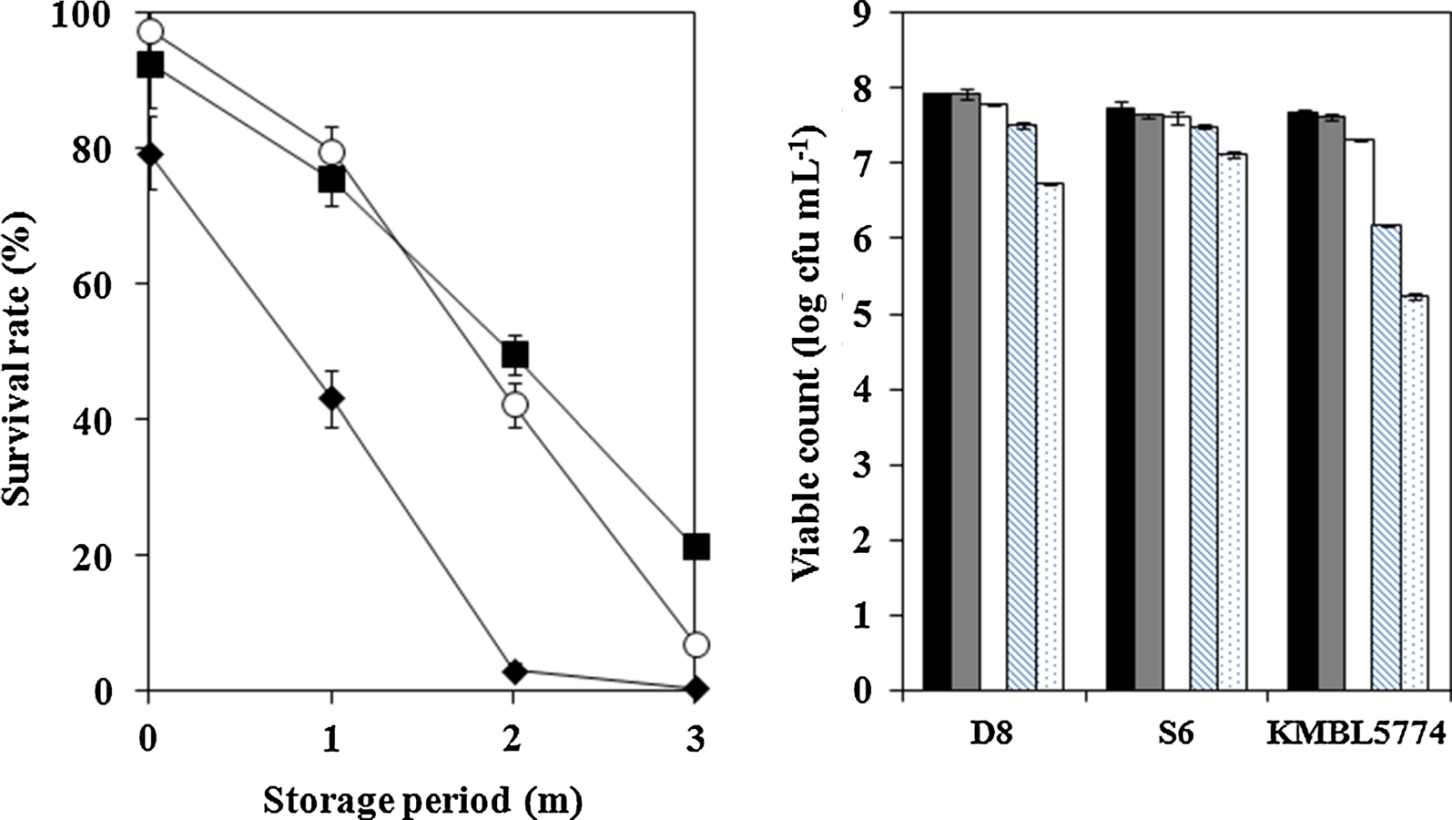
Changes in the survival rate (*left panel*) and viable count (*right panel*) of air-blast dried yeast cells stored at 4 °C for 3 months. *Empty circles*, *filled squares*, and *filled diamonds* in the *left panel* represent the survival rates of *S. cerevisiae* D8, *H. uvarum* S6, and *I. orientalis* KMBL5774, respectively. *Bars* in the histogram (*right panel*) represent viable counts of yeast cells before (*black*) and after (*gray*) air-blast drying and yeasts stored for *1* (*white*), *2* (diagonally patterned), and *3* (*dotted*) months. 10% skim milk and 7 g lactomil were used in all samples. 10% trehalose, 10% sucrose, and 10% glucose were used as protective agents for *S. cerevisiae* D8, *H. uvarum* S6, and *I. orientalis* KMBL5774, respectively. All data are expressed as the mean ± SD (n = 3)

Changes in the survival rate and viable count of air-blast dried yeast cells were investigated in products that had been stored at 4 °C for 3 months (Fig. 4). All samples were prepared based on the optimal conditions determined in the present study. Air-blast dried *S. cerevisiae* D8 and *H. uvarum* S6 continued to show a high survival rate of 42.24 and 49.74% after 2 months, whereas the survival rate of air-blast dried *I. orientalis* KMBL5774 rapidly decreased compared to the other yeast strains and showed a survival rate of only 3.08% after 2 months of storage. After 3 months, the viable count of *S. cerevisiae* D8 and *H. uvarum* S6 decreased to 1.18 and 0.51 log cfu mL^−1^ compared to the viable counts measured immediately after air-blast drying. Therefore, air-blast dried yeast cells of both strains, *S. cerevisiae* D8 and *H. uvarum* S6, have excellent potential as a starter product. On the other hand, although *I. orientalis* KMBL5774 also showed high survival rate immediately after air-blast drying, further study on the long-term storage of *I. orientalis* KMBL5774 is necessary because it showed very low viable count after 3 months of storage (2.38 log reduction).

### Changes in metabolic activities of air-blast dried yeast cells

**Fig. 5:**
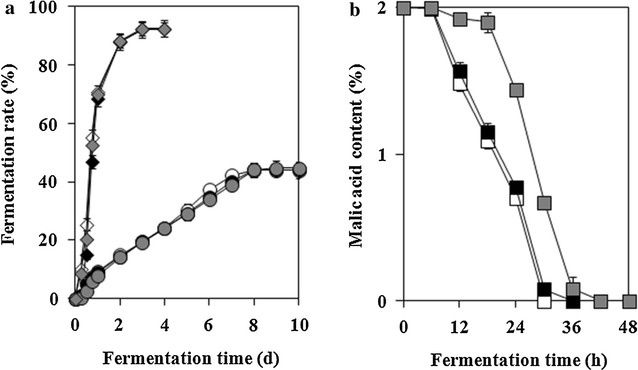
Fermentation rate of air-blast dried *S. cerevisiae* D8 (*diamonds*) and *H. uvarum S6* (*circles*) (**a**) and malic acid content fermented by air-blast dried *I. orientalis* KMBL5774 (*squares*) (**b**). *Empty figures* represent non-dried yeast cells, *filled figures* represent air-blast dried yeast cells just after air-blast drying, and *gray figures* represent air-blast dried yeast cells after 3 months of storage. All data are expressed as the mean ± SD (n = 3)

The metabolic activities of each air-blast dried yeast cell were investigated depending on the storage period (Fig. 5). Glucose fermentation ability of air-blast dried *S. cerevisiae* D8 and *H. uvarum* S6, and malic acid decomposition ability of *I. orientalis* KMBL5774 were examined. All samples were prepared by the optimal manufacturing process based on results obtained in the present study and non-dried yeast cultures were used as control to compare the metabolic activity. Non-dried *S. cerevisiae* D8 decomposed glucose slightly faster compared to both air-blast dried *S. cerevisiae* D8 immediately after drying and after 3 months of storage. However, all samples showed similar fermentability on the second day and completed the fermentation process on third day. Similarly, both air-blast dried *H. uvarum* S6 just after drying and after 3 months of storage showed similar fermentation rates compared to non-dried *H. uvarum* S6. Non-dried *I. orientalis* KMBL5774 initiated and completed malic acid decomposition first, but the duration of malic acid decomposition was not significantly different from that observed for air-blast dried *I. orientalis* KMBL5774 immediately after drying. In case of air-blast dried *I. orientalis* KMBL5774 after 3 months of storage at 4 °C, malic acid degradation was delayed by 12 h compared to that of the control. The results of the metabolic activities of air-blast dried yeast cells after 3 months of storage suggest that *S. cerevisiae* D8 and *H. uvarum* S6 retained their capacities and efficiencies as yeast starters, whereas reduction of the survival rate after long-term storage possibly induced the decrease in the malic acid degradation rate in *I. orientalis* KMBL5774.

## Discussion

In this study, air-blast drying was established as a suitable substitute to conventional drying methods, such as freeze-drying or spray-drying for manufacturing yeast starters for wine. Skim milk, generally used as a protective agent in freeze-drying, was added at 5 and 10% to air-blast dried yeast cells and the addition resulted in the increase in the survival rate of air-blast dried yeast cells in a dose-dependent manner (Fig. 1). Ananta et al. (2005) reported that when 20% reconstituted skim milk added to *Lactobacillus rhamnosus* GG was spray dried at an outlet temperature of 80 °C, its survival rate was measured to be >60%. Abadias et al. (2001a) reported that freeze-dried *Candida sake* showed the highest survival rate of 40% when 10% skim milk and 10% lactose were added as protective agents. Similarly, our study also showed that skim milk had a protective effect on air-blast dried yeast cells.

Four excipients (wheat flour, nuruk, artichoke powder, and lactomil) were evaluated to make an appropriate powdered form of the final product of the air-dried yeast starter (Table 1). According to a study by Beker and Rapoport (1987), 12–13% moisture content is not suitable for storage of yeast, whereas at 8–10% moisture content, yeast retain a remarkable degree of cell viability during storage. Therefore, all samples were air-blast dried until moisture content of each sample was <10%. Drying times for samples with the excipients wheat flour and artichoke were relatively longer than those observed for the other excipients. This result could be attributed to the water or moisture in these excipients, which could lead to increased viscosity due to starch gelatinization and binding to the gluten network in wheat flour (Fessas and Schiraldi 2001), and to the high dietary fiber content in the artichoke (insoluble 18.11% and soluble 26.74%), which could interact with the water held in the capillary structure of the artichoke (Lintas and Capeloni 1988; López et al. 1996). Nuruk, a Korean traditional starter prepared by the natural proliferation of fungi and other microorganisms (Yoo et al. 2011), was also investigated as excipient for yeast starter. Although drying time for nuruk was shorter than wheat flour and artichoke, the physical properties of nuruk as well as that of wheat flour were not suitable for preparing the final starter product because they led to the formation of lumps after air-blast drying (Fig. 2). On the contrary, lactomil (consisting of lactose and maltodextrin) was considered as the most suitable excipient for yeast starter because it yielded a rapid drying time of 2.5 h and the highest yeast cell survival rate and formed a fine powder. Furthermore, the survival rates of air-blast dried yeast cells, based on the amount of lactomil added, were investigated and all samples mixed with 7 g lactomil statistically showed the highest and optimal survival rate (Table 2).

Sugars have been widely used as protective agents due to their low price, chemically innocuous nature, and general utilization in the food industry (Peighambardoust et al. 2011). The protective effects of various sugars on the survival rate of microbial starters such as yeast and bacteria have been determined in the last few decades (Jofré et al. 2014; Lodato et al. 1999; Niu et al. 2016). Our study showed that each strain showed its highest capacity for survival with different optimal protective agents (Table 3). *S. cerevisiae* D8 added to 10% trehalose showed the highest survival rate of 97.54% and most sugar-based protectants (except for fructose) demonstrated excellent protective effects with >90% survival rate. *H. uvarum* S6 showed the highest survival rate (92.59%) when 10% sucrose was added as a protectant and other sugars were also shown to demonstrate notable protective effects compared to no sugar addition. Contrary to *S. cerevisiae* D8 and *H. uvarum* S6, only 10% glucose and 10% fructose remarkably increased the survival rate of *I. orientalis* KMBL5774, whereas other sugars demonstrated protective effects that only slightly increased the survival rate of *I. orientalis* KMBL5774. The morphology of air-blast dried yeast starters was also observed by SEM to confirm the accumulation of yeast cells (Fig. 3). Pereira et al. (2003) reported that 10% trehalose was able to reduce oxidative damage caused by dehydration in *S. cerevisiae* and Garay-Arroyo et al. (2004) reported that *S. cerevisiae* could be easily adapted to various environmental stresses, including oxidative stress, heat shock, freezing shock, osmotic, and ionic stress. A study by Lemetais et al. (2012) on *S. cerevisiae* showed that the plasma membrane is an essential structure for the survival of cells during dehydration by air-drying. In a study on the survival rate of *H. uvarum* lyophilized without cryoprotectant and stored at −80 °C for 0–12 months, the viable counts showed a reduction of 2.47–2.82 log cfu mL^−1^ from the values recorded before freeze-drying (Pietrowski et al. 2015). A study by Kim et al. (2016) reported that *S. cerevisiae* D8, *H. uvarum* S6, and *I. orientalis* KMBL5774 entrapped in 2% Ca-alginate beads by air-blast drying showed 90.67, 90.81, and 87.04% survival rate when 10% skim milk and 10% sugars (sucrose, trehalose, and glucose for *S. cerevisiae* D8, *H. uvarum* S6 and *I. orientalis* KMBL5774, respectively) were used as protectants. Miyamoto-Shinohara et al. (2010) reported that *I. orientalis* had an 8.6 and 28.2% survival rate on freeze-drying and liquid drying, respectively, without any protective agent.

Long-term storage is the most important factor in developing microbial starters for industrial use. In our study, long-term storage effect of air-blast drying on each yeast strain stored at 4 °C for 3 months was investigated (Fig. 4). Air-blast dried *S. cerevisiae* D8 and *H. uvarum* S6 showed 1.18 and 0.51 log reductions, which means that these strains retained a good viable count after 3 months of storage. However, since *I. orientalis* KMBL5774 showed a 2.38 log reduction after 3 months of storage, further study would be needed to improve its storability. Several studies have reported storability based on various drying method and protectants. A study by Miyamoto-Shinohara et al. (2006) reported that freeze-dried *S. cerevisiae* showed 0.010 log reduction per year for 20 years. Zayed and Roos (2004) demonstrated that 4% sucrose, 4% trehalose, and 18% skim milk, used as protective solutions for freeze-dried *Lactobacillus salivarius* maintained the survival rate at 83–85% for 7 weeks of storage. In a study by Gardiner et al. (2000), the survival rate of spray-dried *Lactobacillus paracasei* NFBC 338 grown in 20% reconstituted skim milk was maintained at constant at ~1 × 10^9^ cfu g^−1^ during 2 months of storage at 4 °C, while storage of *L. salivarius* UCC 118 under the same conditions showed 1 log reduction (from 7.2 × 10^7^ to 9.5 × 10^6^ cfu g^−1^).

Fermentation rates of air-blast dried *S. cerevisiae* D8 and *H. uvarum* S6 and the malic acid decomposition ability of air-blast dried *I. orientalis* KMBL5774 after 3 months of storage were analyzed to evaluate their metabolic capacities (Fig. 5). Fermentation rates of *S. cerevisiae* D8 and *H. uvarum* S6 showed no significant difference in all samples. On the other hand, malic acid degradation by air-blast dried *I. orientalis* KMBL5774 after 3 months of storage was delayed by 12 h compared to that of non-dried yeast cells; this result could be attributed to the low survival rate of air-blast dried *I. orientalis* KMBL5774 after 3 months of storage. In a study by Bekatorou et al. (2001), freeze-dried *S. cerevisiae* cells immobilized on gluten pellets showed higher glycolytic activity concerning the fermentation time than free freeze-dried *S. cerevisiae* cells because immobilization increased the viable count of freeze-dried cells. A study by Pietrowski et al. (2015) demonstrated that the maximum fermentation rate of lyophilized *H. uvarum* took longer to achieve than the cryopreserved *H. uvarum* because of reduction in the initial cell population (10^4^ cfu mL^−1^).

In summary, air-blast drying method is a suitable alternative to conventional drying methods for making yeast starter. Yeast cells retained excellent viability after air-blast drying when 10% skim milk and 10% sugars were used as protective agents and 7 g lactomil was used an excipient. The viability and availability of these yeast starter products (except for *I. orientalis* KMBL5774) was supported by the results of long-term storability and metabolic activity. Therefore, our study suggests that the air-blast drying method can contribute to optimal manufacturing processes for microbial starters of industrial value.
